# Optimization in the expression of ASFV proteins for the development of subunit vaccines using poxviruses as delivery vectors

**DOI:** 10.1038/s41598-021-02949-x

**Published:** 2021-12-06

**Authors:** Jaime Lopera-Madrid, Lex G. Medina-Magües, Douglas P. Gladue, Manuel V. Borca, Jorge E. Osorio

**Affiliations:** 1 Department of Pathobiological Sciences, School of Veterinary Medicine, University of Wisconsin-Madison, Madison, WI 53706 USA; 2 Plum Island Animal Disease Center, Agricultural Research Service (ARS), Greenport, NY 11944 USA

**Keywords:** Viral infection, Genetic vectors

## Abstract

African swine fever virus (ASFV) causes a highly contagious hemorrhagic disease that affects domestic pig and Eurasian wild boar populations. To date, no safe and efficacious treatment or vaccine against ASF is available. Nevertheless, there are several reports of protection elicited by experimental vaccines based on live attenuated ASFV and some levels of protection and reduced viremia in other approaches such as DNA, adenovirus, baculovirus, and vaccinia-based vaccines. Current ASF subunit vaccine research focuses mainly on delivering protective antigens and antigen discovery within the ASFV genome. However, due to the complex nature of ASFV, expression vectors need to be optimized to improve their immunogenicity. Therefore, in the present study, we constructed several recombinant MVA vectors to evaluate the efficiency of different promoters and secretory signal sequences in the expression and immunogenicity of the p30 protein from ASFV. Overall, the natural poxvirus PrMVA13.5L promoter induced high levels of both p30 mRNA and specific anti-p30 antibodies in mice. In contrast, the synthetic PrS5E promoter and the S E/L promoter linked to a secretory signal showed lower mRNA levels and antibodies. These findings indicate that promoter selection may be as crucial as the antigen used to develop ASFV subunit vaccines using MVA as the delivery vector.

## Introduction

African swine fever (ASF) is a highly contagious viral disease of swine that leads to high mortality in domestic pigs while asymptomatic in the natural suid reservoir hosts^1,2^. ASF is caused by the African swine fever virus (ASFV), a member of the *Asfarviridae* family. It is the only DNA virus transmitted by arthropods^3^, and to date, all efforts to produce a commercial vaccine against ASF have failed; therefore, prevention relies entirely upon preventing contact between the virus and the susceptible host. Importantly, studies have shown that immunization of pigs with naturally attenuated ASFV strains, viruses attenuated by the passage in cell culture, or by deletions of genes involved in virus virulence can induce protection against related virulent viruses^4–6^. Some obstacles that have hindered an ASF vaccine’s development are the lack of heterologous protection among strains due to the high genetic diversity (22 virus genotypes) and virus complexity (160–175 genes encoded)^3^. A disadvantage of using live attenuated vaccines in the field may be the risk of persistence of the vaccine strain, and the potential presence of residual virulence^7^.

The growth in information regarding ASFV genes’ functions and available genomic sequences from isolates has opened the possibility for the rational design of ASF vaccines. In this regard, some progress has been made in identifying proteins that may induce a protective response. Antibodies against proteins p72 and p54 inhibit virus attachment, while antibodies to protein p30 inhibit virus internalization^8^. Immunization of pigs with a mixture of baculovirus-expressed p54 and p30 proteins, or a chimera of both these proteins, induced neutralizing antibodies and modified the course of the disease; however, a variable degree of protection was obtained^9,10^.

Recently, immunization using a mix of different virus proteins in a variety of immunizing vectors has shown the induction of at least partial protection against the virus challenge suggesting the feasibility of developing subunit vaccines in ASF^11^.

Recombinant poxviruses have become a promising tool for developing new human and veterinary vaccines during the last decades. They provide many advantages as antigen delivery systems, such as inserting large fragments of extra DNA (up to 25 kb), thus allowing the generation of multivalent vaccines by insertion and expression of several transgenes^12^. Importantly, replicating poxvirus vectors has been shown to induce long-lasting immunity and activate humoral and cellular immunity depending on the promoter used to express the antigen^13^. In addition, ASFV and poxvirus shares similarities at the structural level, suggesting that recombinant ASFV proteins may be properly expressed in poxvirus vectors. One of the most advanced poxvirus vaccine vectors is MVA, a non-replicating strain of vaccinia virus that has been evaluated in different animal models as a vaccine candidate against viral, bacterial and parasitic infections^14^. Although MVA undergoes limited or no reproductive replication in mammalian cells, most of the viral genes, including the transgenes, are expressed; thus, the humoral and cellular immunity can be triggered against the recombinant antigens^14^. In the case of ASFV, recent studies have demonstrated the immunogenicity of MVA when used as a viral vector to express ASFV antigens in pigs^15,16^.

In viral vectored vaccines, strong recombinant antigen expression and timing of expression influence the quantity and quality of the immune response. Therefore, natural and synthetic promoters and regulatory sequences have been used in recombinant viral vectors to improve specific-antigen immune responses. For instance, the PrMVA-13.5L promoter, a unique and novel naturally occurring promoter in MVA, is highly conserved across orthopoxviruses. Studies of promoter activity revealed that the PrMVA13.5L produced higher protein levels early during infection than commonly used promoters^17^. Early studies on vaccinia virus early and late (E/L) promoters led to the design of one of the most common promoters used in poxvirus-based vaccines, the short synthetic early/late (S E/L)^18^. However, novel hybrid early/late promoters such as PrS5E and pHyb have been proposed to improve antigens’ expression levels and induce strong antigen-specific immune responses^18,19^. Similarly, the inclusion of molecular elements such as internal ribosomal entry site (IRES) and the secretory signals tPA (tissue plasminogen activator) and C13L have been shown to enhance antigens’ expression levels significantly^20^.

In this study, we constructed and characterized five recombinant MVA viruses to evaluate the efficiency of different promoters and secretory signal sequences in the expression and immunogenicity of a well-characterized ASFV antigen, the early transmembrane p30 protein. Five different promoters were evaluated: a natural poxvirus promoter (PrMVA13.5L), three synthetic promoters (pHyb, PrS5E, and S E/L), and a synthetic promoter followed by a secretory signal (S E/L-C13L). Then, we compared the strength of these promoters by measuring p30 mRNA expression levels in DF-1 and Vero cells and the fluorescence intensity as an indirect measurement of their activity. When we evaluated the immunogenicity of three of these recombinant viruses in mice, we found that the MVA-PrMVA13.5L-p30 virus induced the highest levels of anti-p30 antibodies after repeat vaccinations, making it an appealing promoter to improve the immunogenicity of MVA-based vaccines. Our results may provide important information to develop ASFV vaccine candidates using MVA as the delivery vector and warrant future investigations in pigs.

## Results

### Characterization of recombinant MVA-p30 viruses

**Figure 1:**
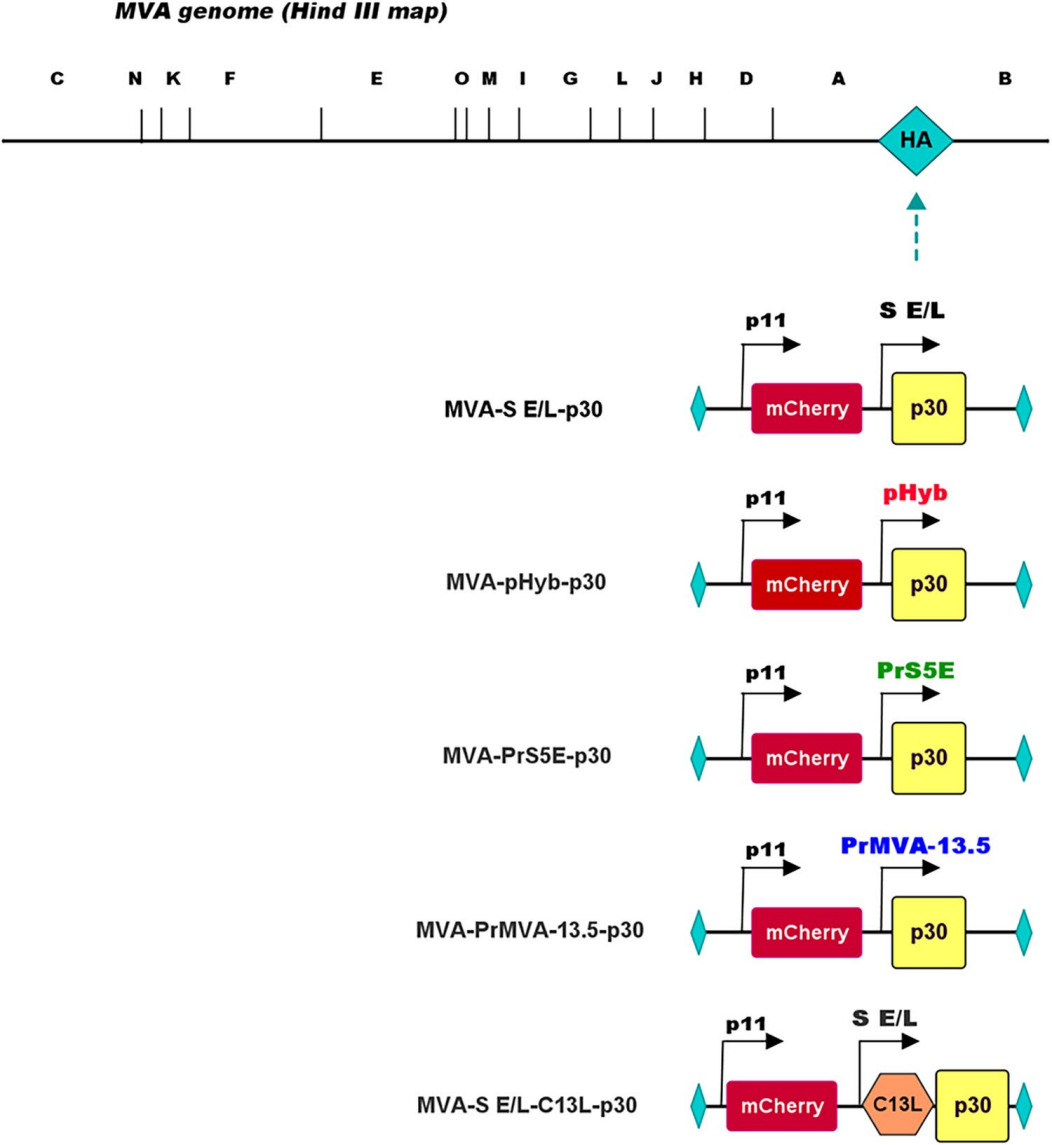
Generation and characterization of MVA-p30 viruses. Diagram of the MVA genome and DNA fragments used for homologous recombination to generate the recombinant MVA viruses. *Hind III* restriction endonuclease sites within the genome of MVA and the HA gene are indicated. Each expression cassette containing the p30 gene under the control of either the S E/L, pHyb, PrS5E, or PrMVA-13.5L promoters, and the S E/L promoter in frame with the C13L secretory signal are depicted. Also included in each cassette is the mCherry gene under the control of the p11 promoter and flanking regions to the HA gene, where the expression cassettes were inserted into the MVA genome.

In this study, we constructed and characterized the antigen expression levels and immunogenicity of five recombinant MVA viruses expressing the p30 antigen from ASFV. Four of these constructs tested the importance of the promoter selection (PrMVA-13.5L, pHyb, PrS5E, and S E/L, and the fifth one evaluated the use of the C13L secretory signal in combination with the S E/L promoter (Fig. 1). The PrMVA13.5L is a native poxvirus promoter classified as strong immediate-early that can induce both antigen-specific antibodies and T cell responses after repeated vaccinations^17^. The pHyb is a hybrid promoter that contains both early and late elements from the p7.5 promoter and the A-type inclusion (ATI) promoter, respectively^19^. Similarly, PrS5E is a novel synthetic promoter containing several early and late elements^17^. S E/L is a compact and synthetic promoter with overlapped early and late regulatory elements^18^. The C13L is a secretory signal from the vaccinia virus that has been used in influenza vaccine candidates that induced strong antibody titers and protection in mice^20^.

The genetic homogeneity of each of the final virus constructs was analyzed by PCR. DF-1 cells seeded on 6-well plates were infected with each recombinant virus at an MOI of 1 for 1 h. At 24 h post-infection (p.i.), viral DNAs were purified and used as templates for PCR amplification by primers specific for the HA gene of MVA (flanking regions). The presence of the mCherry and p30 genes with the corresponding promoter was confirmed in all recombinant MVA viruses by bands at the expected sizes, indicating the genetic stability of the viral constructs throughout all plaque selection rounds. Plasmid DNAs of each cassette were included in the PCR reactions as positive controls. Also, DNA sequencing confirmed that no genetic alterations were present in the final viral stocks (data not shown).

### Characterization of p30 mRNA and protein expression

**Figure 2:**
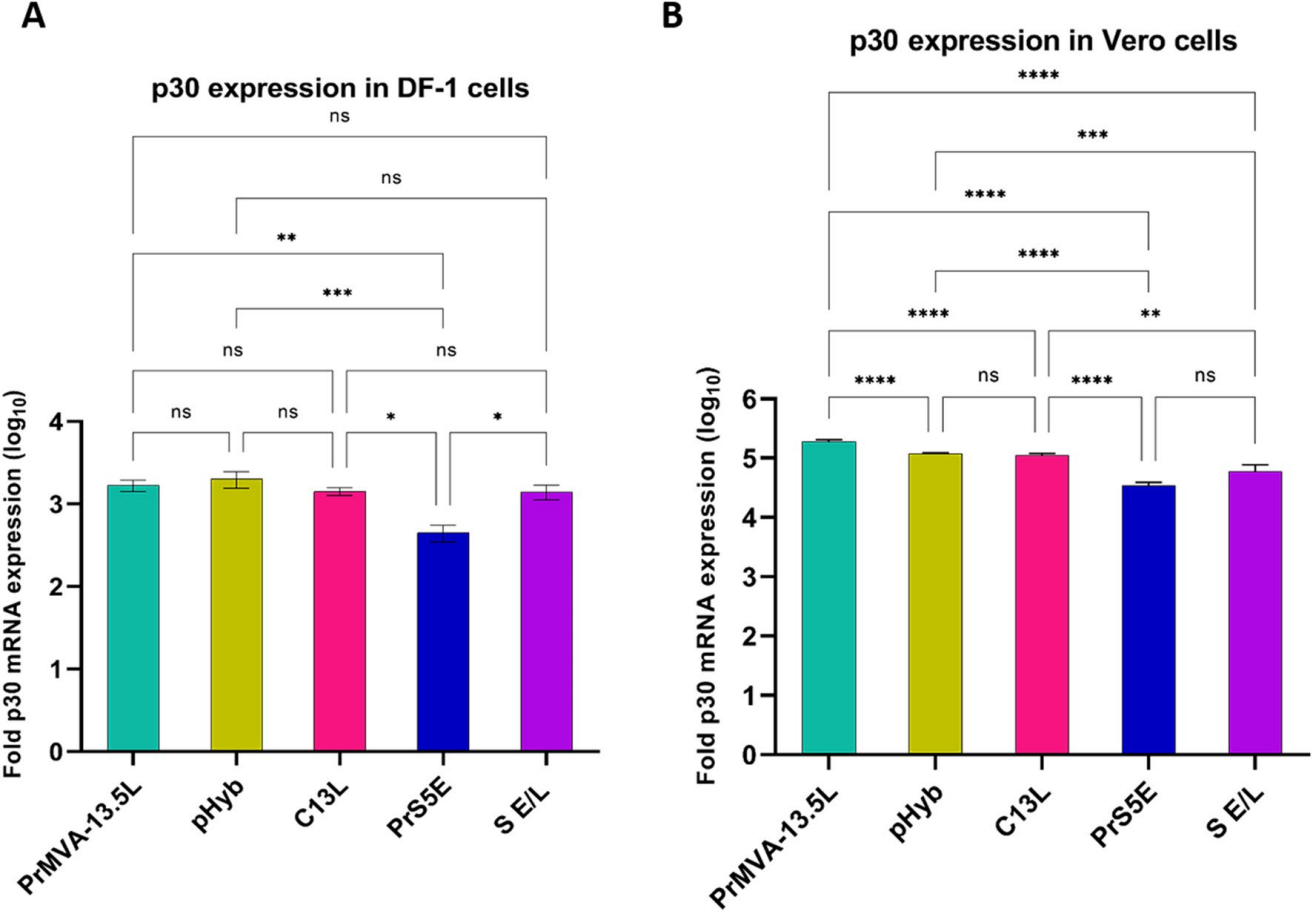
Expression levels of p30 were analyzed at the mRNA level. DF-1 and Vero cells were infected with the indicated MVA-p30 virus, and after 24 h, total RNA was isolated and reverse transcribed. (**A**) indicates mRNAs from DF-1 cells and (**B**) from Vero cells. Fold p30 expression levels were calculated relative to the cells infected with MVA-GFP control and after normalization to the β-actin gene. Sample reactions, including MVA-GFP, were performed in triplicate and two independent repetitions. Statistically significant differences between MVA-p30 viruses are shown (P < 0.05, one-way ANOVA with Tukey’s multiple comparison test). For DF-1 cells: *P < 0.01; **P < 0.002; ***P < 0.0003. For Vero cells: **P < 0.0012; ***P < 0.0004; *****P < 0.0001.

The expression levels of p30 mRNA were assessed for each of the MVA-p30 constructs in infected DF-1 and Vero cells using qRT-PCR (Fig. 2). Differences in p30 mRNA synthesis levels were observed, indicating that promoter selection and secretory signal were important in recombinant vaccine design. In DF-1 cells, the highest levels of mRNA synthesis were observed in constructs containing the pHyb, PrMVA-13.5L, S E/L-C13L, and S E/L, respectively (Fig. 2A). In contrast, the PrS5E promoter showed lower expression levels than the other promoters. In Vero cells, the messenger RNA level produced by the PrMVA-13.5L promoter was higher than those observed for pHyb, S E/L-C13L, PrS5E, and S E/L (P < 0.0001). Additionally, a significant difference was observed in mRNA levels induced by pHyb compared to PrS5E (P < 0.0001) and S E/L (P < 0.0004). Similarly, the S E/L-C13L construct induced higher levels of p30 mRNA when compared to PrS5E (P < 0.0001) and S E/L (P < 0.0012) (Fig. 2B). Interestingly, all constructs showed higher levels of p30 mRNA in Vero cells than in DF-1 cells.

**Figure 3:**
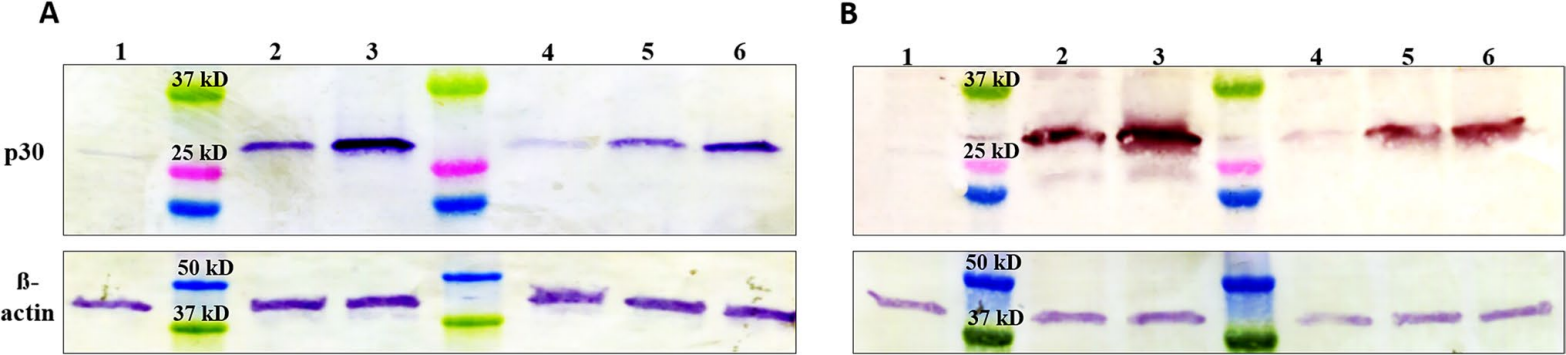
Detection of p30 protein expression by immunoblotting assay. Monolayers of DF-1 and Vero cells were infected with each MVA-p30 virus at an MOI of 5, and then total protein extraction was performed at 24 h p.i., and extracted proteins were subjected to SDS-PAGE followed by western blot analysis. (**A**) indicates proteins from DF-1 cells and (**B**) from Vero cells. The order is as follows, MVA-GFP control (1), PrMVA-13.5 (2), pHyb (3), S E/L-C13L (4), PrS5E (5), and S E/L (6). A parallel blot incubated with a ß-actin specific monoclonal antibody served as loading control. Molecular masses of marker proteins are indicated. Full-length blots are presented in Supplementary Fig. 1.

The expression of p30 antigen by each recombinant virus was monitored by western blot and immunofluorescence assays. Although we observed differences in protein production compared to the p30 mRNA levels, the p30 protein was detected at 24 h p.i. in cell pellet lysates from all construct infected cells, but not in MVA-GFP infected cells (Fig. 3).

The blotted membranes were subjected to imaging analysis to quantify the intensity of the p30 protein from each construct, and the intensity of ß-actin protein was used to obtain the relative quantification of the p30 protein (Fig. 3). In particular, a high-intensity value corresponding to p30 was observed in cells infected with the pHyb construct, followed by S E/L, PrMVA-13.5L, and PrS5E infected DF-1 cells. In contrast, a low intensity value was detected in the S E/L-C13L infected cells (Fig. 3A) (Table 1). In Vero cells, a similar trend compared to DF-1 was observed as the highest intensity value was detected from the pHyb construct while the lowest was from the S E/L-C13L infected cells. Unlike DF-1 cells, the PrMVA-13.5L construct showed a higher band intensity than S E/L but very close to the PrS5E value (Fig. 3B). Interestingly, in this mammalian cell line, the band intensity from the PrS5E construct was slightly higher than the band from the S E/L construct (Table 1).

**Table 1:** Band intensities of p30 protein counted in western blot images.

Construct		PrMVA-13.5L	pHyb	S E/L-C13L	PrS5E	S E/L	MVA control
DF-1 cells	Band intensity	22,784.59	52,146.44	5950.88	19,209.73	43,338.52	1.28
Vero cells		16,091.01	22,410.39	2670.70	16,036.79	15,098.51	1.04

Scanned membranes from immunoblots of DF-1 and Vero cells were processed using the ImageJ software. Bands from each construct were selected and quantified to obtain histograms. The acquired data was normalized using ß-actin to obtain the relative quantification of the p30 protein.

**Figure 4:**
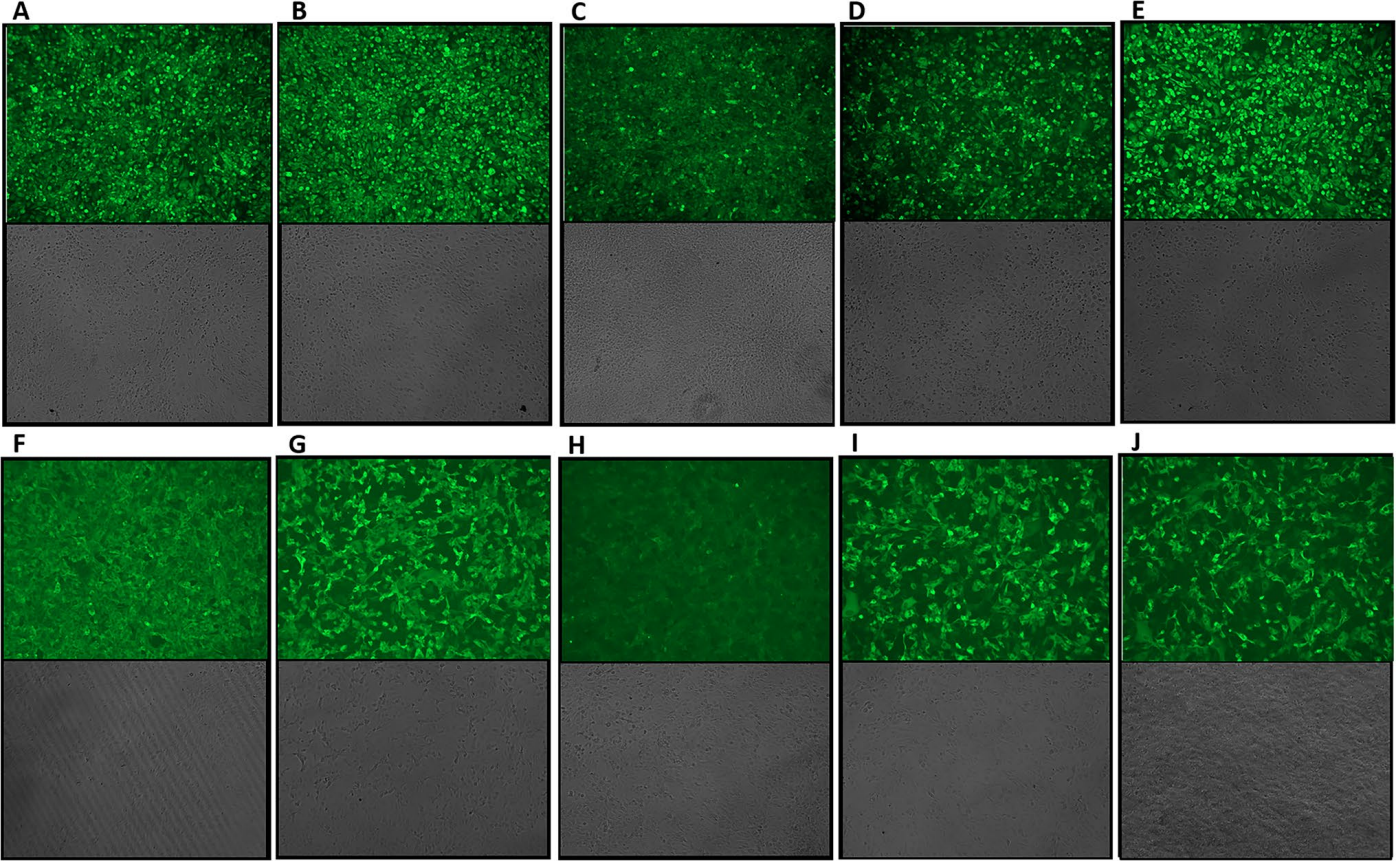
Detection of p30 protein expression by immunofluorescence assay. Monolayers of DF-1 and Vero cells were infected with each MVA-p30 virus at an MOI of 1, and processed 24 h after infection. All images were acquired using a 10X objective and a green filter set at 70% intensity. Row A to E corresponds to DF-1 cells, and row F to J to Vero cells. Each column corresponds to: (**A**,**F**) MVA-PrMVA-13.5L-p30, (**B**,**G**) MVA-pHyb-p30, (**C**,**H**) MVA-S E/L-C13L-p30, (**D**,**I**) MVA-PrS5E-p30, and (**E**,**J**) MVA-S E/L-p30. Bright-field images are also included.

Levels of p30 protein were confirmed in DF-1 and Vero cells using immunofluorescence assay. In DF-cells, quantification of the immunofluorescence images from each construct indicated a correlation with the western blot analysis (Fig. 4). More specifically, the pHyb construct produced the highest mean of fluorescent particles, followed by S E/L, PrMVA-13.5L, PrS5E, and S E/L-C13L constructs (Table 2). Fluorescence intensity and band intensity measured in Vero cells correlated as the pHyb produced the highest mean of fluorescent value followed by PrMVA-13.5L, PrS5E, and then S E/L constructs. However, similar to DF-1 cells, the S E/L-C13L construct produced the lowest fluorescence value.

**Table 2:** Mean of fluorescence particles counted in immunofluorescence images.

Construct		PrMVA-13.5L	pHyb	S E/L-C13L	PrS5E	S E/L	MVA control
DF-1 cells	Mean fluorescence values	167.8	186.6	136.9	144.3	181.9	24.678
Vero cells		165.8	168.8	109.1	150.501	145.466	10.586

DF-1 and Vero cells infected with each construct were photographed at 10X objective (70% brightness), and the mean numbers of cells expressing p30 protein were calculated using the ImageJ software.

### The activity of the natural and synthetic promoters also impacts the expression of mCherry protein

Unexpectedly, we observed that each of the recombinant MVA viruses exhibited differences in the expression of the mCherry protein, as images acquired using a 4X objective showed evident variations in the fluorescence intensity between the viruses even though infections with each recombinant virus were performed at the same MOI. This phenomenon was not related to variability in viral replication as we did not observe differences in the in vitro growth of each virus (data not shown). Therefore, to examine whether the activity of each promoter expressing the p30 protein also affects the activity of the p11 promoter and thus, the expression of the mCherry protein (Fig. 1), we infected DF-1 and Vero cells with each recombinant virus at an MOI of 1, and 24 h p.i., we acquired images using a red filter (Texas red) and a 4X magnification objective, and also collected total RNA from infected cells to measure mCherry mRNA synthesis.

**Figure 5:**
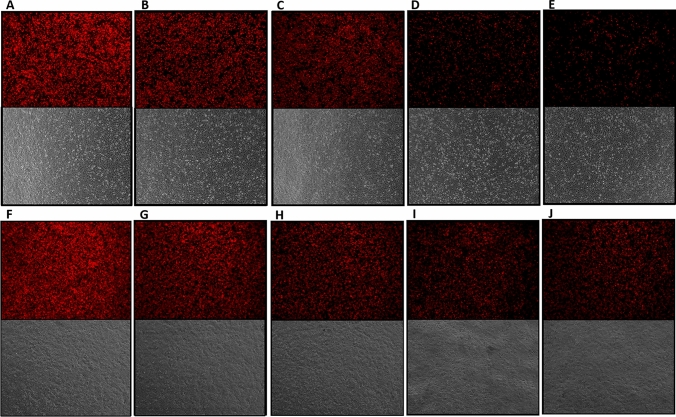
Comparison of fluorescence intensity of recombinant MVA viruses. DF-1 and Vero cells were infected with each recombinant virus at an MOI of 1, and at 24 h p.i., images were acquired using a 4 × objective and a Texas red filter set at 100% intensity. Row A to E corresponds to DF-1 cells, and row F to J to Vero cells. Each column corresponds to: (**A**,**F**) MVA-PrMVA-13.5L-p30, (**B**,**G**) MVA-pHyb-p30, (**C**,**H**) MVA-S E/L-C13L-p30, (**D**,**I**) MVA-PrS5E-p30, and (**E**,**J**) MVA-S E/L-p30. Bright-field images are also included.

In both DF-1 and Vero cells, the recombinant MVA bearing the PrMVA-13.5L promoter showed the highest level of fluorescence intensity (Fig. 5A,F); similarly, the pHyb promoter also showed a high fluorescence intensity (Fig. 5B,G), but it was slightly less intense than the PrMVA-13.5L. Interestingly, the combination of the S E/L promoter and the C13L secretory signal resulted in a significant increase in fluorescence levels (Fig. 5C,H) compared to the expression under the S E/L promoter alone (Fig. 5E,J), which along with the PrS5E (Fig. 5D,I), produced the lowest levels of fluorescence.

**Figure 6:**
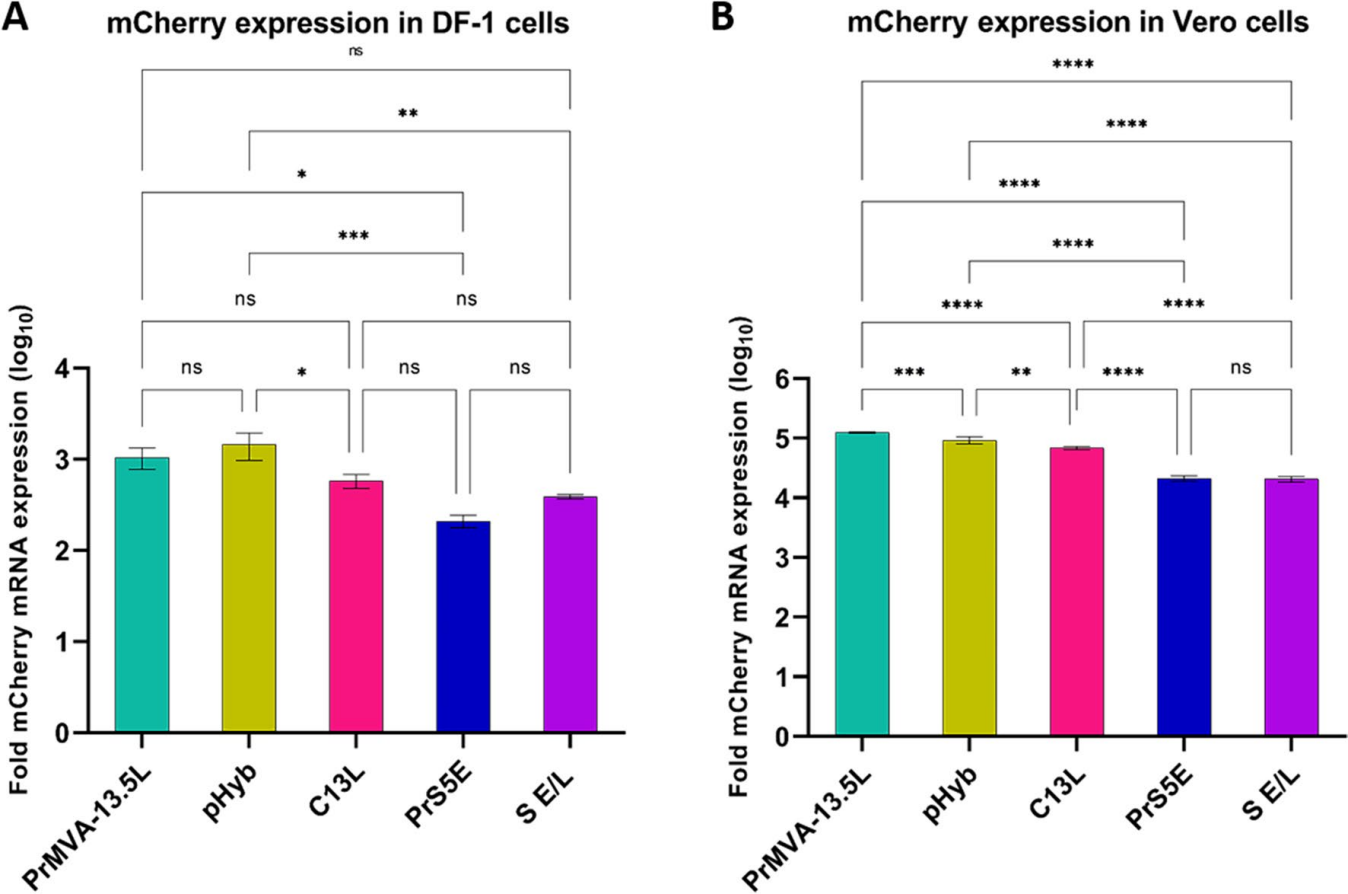
Expression of mCherry mRNA. DF-1 and Vero cells were infected for 24 h with each recombinant virus. After total RNA was isolated and reverse transcribed, the fold expression of mCherry was calculated relative to the cells infected with MVA-GFP control and normalized to the β-actin gene. All reactions were performed in triplicate and two independent repetitions. Statistically significant differences between constructs are shown (P < 0.05, one-way ANOVA with Tukey’s multiple comparison test). For DF-1 cells: *P < 0.01; **P < 0.003; ***P < 0.001. For Vero cells: **P < 0.004; ***P < 0.0006; ****P < 0.0001.

Afterward, we assessed the levels of mCherry mRNA for each construct in infected DF-1 and Vero cells using qRT-PCR (Fig. 6). In DF-1 cells, both PrMVA-13.5L and pHyb constructs induced the highest levels of mCherry mRNA, but no significant difference was observed. A difference was obtained when comparing the PrMVA-13.5L to the PrS5E (P < 0.01) construct. For pHyb, differences were observed when compared to S E/L-C13L (P < 0.01), PrS5E (P < 0.001), and S E/L (P < 0.003) constructs. No differences were observed between S E/L-C13L, PrS5E, and S E/L constructs (Fig. 6A).

In agreement with the fluorescence intensity obtained in Vero cells, the recombinant MVA containing the PrMVA-13.5L promoter induced the highest levels of mCherry mRNA compared to pHyb (P < 0.0006), S E/L-C13L (P < 0.0001), PrS5E (P < 0.0001), and S E/L (P < 0.0001). The pHyb also induced higher levels of mRNA when compared to S E/L-C13L (P < 0.004), PrS5E (P < 0.0001), and S E/L (P < 0.0001). Additional differences were also observed between the S E/L-C13L when compared to PrS5E (P < 0.0001) and S E/L (P < 0.0001). No difference was detected between the PrS5E and S E/L constructs (Fig. 6B). Similar to the p30 mRNA levels, all constructs produced higher levels of mCherry mRNA in Vero cells than in DF-1 cells.

### Immunogenicity in BALB/c mice

**Figure 7:**
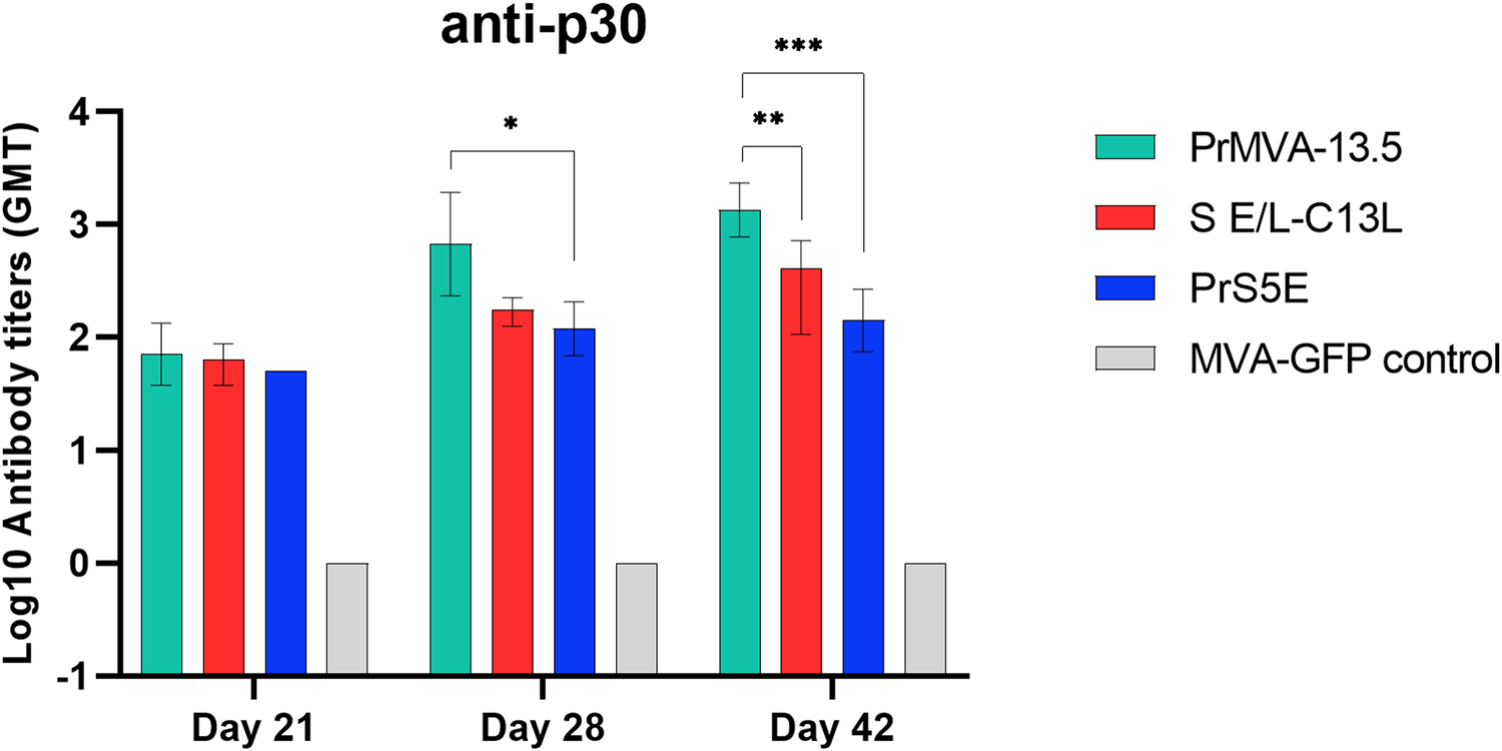
Characterization of serum samples obtained from mice vaccinated with MVA-p30 constructs. BALB/c mice were primed and boosted (days 21 and 35) with either MVA-PrMVA-13.5L-p30, MVA-S E/L-C13L-p30, MVA-PrS5E-p30, or MVA-GFP. Purified p30 protein (baculovirus-expressed) were used as coating antigen for ELISA assays. Detection of specific anti-p30 antibodies at days 21, 28, and 42 p.i. for all animal groups are shown (n = 4 mice per group). Statistically significant differences between antibody titers are shown (P < 0.05, one-way ANOVA with Tukey’s multiple comparison test) *P < 0.05; **P < 0.005; ***P < 0.0005.

An immunization study was conducted in mice to evaluate the induction of specific anti-p30 antibodies by some of the constructs. We tested the PrMVA-13.5L, the S E/L-C13L, and the PrS5E promoters. Based on the western blot and immunofluorescence assays, the S E/L-C13L construct produced low levels of p30 protein in both DF-1 and Vero cells; therefore, this construct was chosen to determine whether these low levels of expression also translate into a low level of anti-p30 antibodies. Although the pHyb showed higher levels of p30 protein expression in Vero cells than both the PrMVA-13.5L and PrS5E constructs, it is interesting to evaluate in vivo two very different promoters such as the full naturally occurring MVA promoter, PrMVA-13.5L, and a synthetic hybrid promoter, PrS5E, which showed low levels of p30 mRNA but higher levels of protein production in this mammalian cell line than the S E/L construct. To carry out the study, mice were primed and then boosted at days 21 and 35, and serum samples were collected at 0, 21, 28, and 42 p.v. Although three weeks after the first immunization, all promoter constructs induced similar p30 antibody titers, a boost effect in these titers was detected after a second immunization, day 28 p.i., and differences were observed in particular with the PrMVA-13.5L compared to the rest of the promoters analyzed (Fig. 7). This promoter induced higher levels of anti-p30 antibodies than the S E/L-C13L promoter and a significant difference compared to the PrS5E promoter (P < 0.05). This difference was more significant after the third immunization as titters induced by the PrMVA-13.5L construct were significantly higher than the S E/L-C13L and PrS5E promoters (P < 0.005; P < 0.0005, respectively).

## Discussion

MVA has demonstrated to be a highly immunogenic antigen-expressing vector with an excellent safety profile; however, many studies using MVA-based vaccine candidates, including ASFV, have mainly focused on selecting potentially protective antigens than molecular regulators that may improve their level of expression.

Despite the safety and immunogenicity profile exhibited by MVA, further improvements of the magnitude, breadth, and durability of the immune responses against expressed antigens should be pursued. One way of improving MVA immunogenicity is using promoters that can enhance the generation of antigen-specific immune responses. In this regard, it has been demonstrated that both timing and antigen expression levels can impact immune responses^21^. Consequently, it is critical that efforts towards developing new poxvirus-based ASFV vaccine candidates focus on using the best available promoters to improve immune responses. In the case of vaccines based on viral vectors expressing multiple ASFV antigens, using the same promoter in a single expression cassette or within the same vector may produce genetic instability due to recombination events between the promoters^22^. Therefore, the next vaccine candidates based on viral vectors co-expressing multiple ASFV genes may require the use of a broader selection of poxvirus promoters that could be used without compromising their genetic stability.

Accordingly, in this study, we evaluated several promoters that may be used to optimize MVA-vectored ASFV vaccine candidates. Toward that aim, we constructed different recombinant MVA viruses expressing the p30 protein under the control of the promoters PrMVA-13.5L, pHyb, PrS5E, S E/L, and a construct containing the S E/L promoter in frame with the C13L secretory signal. The expression kinetics and immunogenicity of these recombinant viruses were assessed in vitro, and their immunogenicity was tested in mice.

We observed a difference in the synthesis of mCherry mRNA and thus in the fluorescence intensity in both DF-1 and Vero cells infected with each construct. All constructs contain the mCherry gene under the control of the vaccinia p11 promoter, which is located upstream and relatively near each of the p30 promoters, around 711 base pairs (Fig. 1). In this regard, studies in human cells have demonstrated that distant genes could influence each other and transcribed cooperatively via promoter-promoter interactions, and that this interaction may be facilitated by chromatin remodeling and bridging proteins^23^. Although these promoter-promoter interactions have been described in mammalian cells, it may be possible that this phenomenon occurs in the context of MVA infection. Specifically, both p11 and PrMVA-13.5L are naturally occurring vaccinia virus promoters, and cells infected with the PrMVA-13.5L construct showed the highest fluorescence levels in Vero cells. On the other hand, the pHyb, PrS5E, and S E/L are synthetic hybrid promoters that contain early and late promoter elements from the vaccinia virus; however, their distribution and position within each promoter may favor or affect any interaction with p11. To test whether a positive promoter-promoter interaction occurs between promoters with either vaccinia virus origin or containing elements from this virus, a similar approach could be implemented where expression cassettes containing both non-vaccinia and vaccinia promoters are used to evaluate the production of the mCherry protein by p11.

For the development of ASFV subunit vaccines, these interactions may be of great importance in designing vaccine candidates expressing multiple recombinant antigens. For instance, p11, PrMVA-13.5L, and pHyb promoters can be included in an expression cassette containing high immunogenic ASFV antigens. These interacting and strong promoters may act and transcribe cooperatively from this single cassette, thus enhancing all antigens' expression levels. Additionally, this strategy may help prevent potential genetic instability when using the same promoter within an expression cassette due to recombination events between the promoters.

We assessed the p30 mRNA expression levels produced by all constructs in DF-1 and Vero cells. Interestingly, the levels of both p30 and mCherry mRNA were higher in Vero cells than in DF-1 cells. Although MVA was developed by serial passage in chicken fibroblast tissue culture and is replication-deficient in cells of mammalian origin, it is able to efficiently express viral and recombinant genes as seen here in this study. In terms of ASFV vaccine development, it is encouraging that recombinant MVAs could induce high levels of transgene expression in mammalian cells.

It is also interesting that the naturally occurring vaccinia promoter PrMVA-13.5L showed an increase in both p30 and mCherry mRNA levels in Vero cells compared to DF-1 cells, in which it was expected to induce higher or similar levels than the pHyb promoter. Nonetheless, this promoter, despite inducing higher levels of p30 mRNA in Vero cells when compared to the pHyb, generated less protein in the infected cells. This difference in transcriptional strength could be related to the two unique and potent early elements in this promoter that potentiate its kinetics of transcription, but the discrepancy between this transcriptional strength and p30 protein production should be addressed in future studies to better define its in vitro activity. Even though this strength was observed at an mRNA level, this feature makes the PrMVA-13.5L a promising promoter to be used in ASFV vaccine candidates.

In contrast, the S E/L, which is classified as strong, compact promoter containing early and late elements, consistently induced low levels of p30 and mCherry mRNA in Vero cells. Remarkably, the S E/L-C13L construct, containing the same S E/L promoter, produced similar p30 mRNA levels to pHyb and higher than S E/L in Vero cells. Because the same promoter is present in both constructs, it is possible that the inclusion of the C13L secretory signal, in the context of p30, might boost/stabilize the production of mRNA by a mechanism that remains to be determined. Additionally, future studies should explore combinations of the C13L secretory signal with the promoters evaluated in this study, including combinations of promoters with another secretory signal such as tPA. These combinations may help determine whether the increase of p30 mRNA levels is restricted to the S E/L-C13L combination, or it can be observed with other promoters/secretory signals.

In terms of p30 protein production in DF-1 and Vero cells, the S E/L-C13L construct produced high levels of p30 mRNA, similar to pHyb. However, the low-intensity band from the cell pellet and fluorescence value observed in cells infected with this construct could be related to the protein processing and secretion directed by C13L. Therefore, we evaluated the supernatant fractions either concentrated by ultrafiltration using a commercial membrane protein purification system or unfiltered to detect the secretion of the p30 protein. Unfortunately, we could not detect the target protein from any of the constructs even when we modified some steps of the immunoblotting protocol to increase the sensibility of the assay. In the case of the S E/L-C13L, the p30 protein processing by the activity of the C13L secretory signal may be responsible for the difficulty in detecting this protein in both the cell pellet and supernatant fraction.

Although the PrS5E construct induced lower levels of p30 protein in DF-1 cells compared to the PrMVA-13.5L and S E/L, the protein level increased in Vero cells as observed in the immunofluorescence assay. One possible explanation for this difference is that in DF-cells, the position and activity of the multiple elements that make up the PrS5E promoter could negatively affect its strength and thus the expression of p30. Similarly, the reduction in Vero cells of the p30 expression by the S E/L promoter could be due to the position and activity of its overlapping early and late elements.

For the development of ASFV subunit vaccines in swine, the PrMVA-13.5L and pHyb promoters induced in the mammalian cell line high levels of p30 mRNA, p30 protein, and mCherry mRNA via a potential promoter-promoter interaction with p11. These promoters may significantly impact the design of multi-antigen expression cassettes that also incorporate highly immunogenic ASFV antigens.

A comparative immunogenicity study in mice indicated a difference in the antibody response to p30. In particular, a positive relationship between the number of vaccinations and immunogenicity was observed for PrMVA-13.5L at days 28 and 42 p.v. (first and second boost, respectively), as this construct showed a 3 to fourfold difference in p30 antibody titers compared with S E/L-C13L. Similarly, a difference of 5 to eightfold of antibody response was seen between PrMVA-13.5L and PrS5E promoters, which is consistent with the p30 mRNA and protein levels observed in PrMVA-13.5L and PrS5E-infected cells. In MVA, strong CD8 T cell responses have been shown to correlate with high early antigen expression, followed by potent recall responses after repeat boosts^24^. In this regard, it is possible that the timing of antigen expression is also responsible for the difference of p30 antibody levels between the promoters. In particular, PrMVA13.5-L has two strong early core elements, whereas PrS5E contains a tandem of five early elements from the p7.5 k promoter, and S E/L-C13L only has an early element. However, the activity of this tandem and the single early element may be compromised by their position relative to the open reading frame of the p30 gene and thus may not function optimally.

Recent studies have evaluated recombinant poxviruses as delivery vectors in ASFV vaccine candidates. One approach evaluated a heterologous prime/boost vaccination using a pool of antigens in a DNA-prime/vaccinia-boosting regime. After challenge with a lethal dose of ASFV Georgia 2007/1, all animals developed acute ASF, but a reduction in viral genome levels was observed^25^. In another study, a similar heterologous vaccination was evaluated using eighteen antigens delivered in an adenovirus-priming/MVA-boosting regime; however, animals were not protected after challenge, but reduced levels of virus loads were achieved^26^. The poxvirus vectors evaluated in the above-described studies used the S E/L promoter to express the ASFV antigens; therefore, it is tempting to speculate that using a strong promoter like PrMVA-13.5L or pHyb in those studies might have strengthened the outcome in terms of immunity and viremia levels.

In summary, the results of the present study suggest that promoter selection, in conjunction with the expression of highly immunogenic antigens, may be crucial for the development of ASFV subunit vaccines using MVA as the delivery vector. Our results have implications for the design of poxvirus-based ASFV vaccines that can be used in homologous or heterologous vaccination regimes. Future studies will be directed at evaluating T cell responses induced by each promoter and whether they correlate with the humoral immune responses described here. Additionally, the activity of these promoters will be assessed in the context of other ASFV antigens, such as p72, to determine if potential differences in immune responses may be related to the intrinsic immunogenicity of the antigen or the strength of the promoters.

## Material and methods

### Cells and viruses

DF-1 cells (ATCC CRL-12203) and African Green monkey (*Cercopithecus aethiops*) kidney epithelial cells (ATCC #CCL-18) were obtained from ATTC (American Type Culture Collection, Manassas, VA) and maintained in Dulbecco’s modified Eagle medium (DMEM; Gibco, Carlsbad, CA) supplemented with 10% fetal bovine serum (FBS), 100 U/ml of penicillin, 100 µg/ml of streptomycin, and 2.5 µg/ml amphotericin B, and incubated at 37 °C in 5% CO2. MVA virus used in these studies was obtained through BEI Resources (NIAID, NIH, ref # NR-727). A recombinant MVA virus expressing GFP (MVA-GFP) was based on the wild-type MVA, and its construction has been previously described^20,27^.

### Generation of recombinant viruses

Five recombinant MVA viruses were constructed. Four of them were bearing the ASFV p30 gene being expressed under the control of different promoters such as: (1) poxvirus PrMVA-13.5L (MVA-PrMVA-13.5L-p30), (2) pHyb (MVA-pHyb-p30), (3) S E/L (MVA-S E/L-p30), and (4) PrS5E (MVA-PrS5E-p30). A fifth MVA recombinant contained the ASFV p30 gene under the control of the S E/L promoter and in-frame with the vaccinia secretory signal C13L (MVA- S E/L-C13L-p30) (Fig. 1). DNA cassettes containing these sequences, the mCherry gene under the control of a late p11 promoter, and flanking sequences from the MVA hemagglutinin gene (HA) were synthesized^28^. Expression of the mCherry protein allows for visual-based selection and permits an easy distinction between recombinant (red) and wild-type (green) viruses.

All cassettes were PCR amplified using Phusion High-Fidelity DNA polymerase (New England Biolabs, # M0530S, Ipswich, MA), and then used for homologous recombination. Recombinant MVA viruses were generated as described previously^29,30^. Briefly, DF-1 cells were seeded into six-well plates the day before transfection and then infected at a multiplicity of infection (MOI) of 0.05 PFU/cell for 1.5 h (h) with wild-type MVA-GFP virus. Following the manufacturer's protocol, cells were then transfected with appropriate PCR fragments using FuGENE HD (Roche Diagnostics, # E2311, Indianapolis, IN). At 48–72 h post-transfection, monolayers were harvested, centrifuged at 500×*g* for 5 min (min) at 4 °C, and cells were disrupted by freeze–thaw (3 times) followed by sonication (2 times for 15 s using a cup sonicator). The disrupted cell extracts were plated again onto fresh DF-1 cells and overlaid with 1% agarose. After 48–72 h, virus-generated plaques expressing mCherry were picked into 500 μL media with a sterile filter pipette tip, then subjected to freeze–thaw, sonication (as described above), and plated to continue passaging. Plaques were passaged until no wild-type (GFP expressing) virus was observed, at which point PCR was performed to confirm the presence of only the recombinant virus^31^. Viral DNA was extracted using the quick-DNA Viral kit from Zymo Research (Zymo Research, # D3015, Irvine, CA), and PCRs were then performed using Phusion High-Fidelity DNA polymerase with specific primers that bind to the flank sequences (HA gene).

High titer virus stocks were produced and further characterized by PCR and DNA sequencing to ensure genetic homogeneity and stability.

### In vitro characterization of recombinant MVA viruses

#### p30 and mCherry mRNA expression analysis in DF-1 and Vero cells using a two-step quantitative reverse transcription PCR (qRT-PCR)

Briefly, DF-1 and Vero cells were infected with an MOI of 5 PFU/cell for 24 h at 37 °C and 5% CO_2_. Infected cells were collected, and RNA was extracted using Direct-zol RNA MicroPrep (Zymo Research, #R2062). One nanogram of RNA was used as the template for the reverse transcription into complementary DNA (cDNA). cDNA was produced using Verso cDNA Synthesis Kit (Thermo Fisher Scientific, #AB1453A, Waltham, MA), and anchored oligo dT was used for priming. The PrimeTime® Gene Expression Master Mix (IDTdna, #1055770), a final concentration of 0.5 mM for each primer, and 0.25 mM of probe synthetized by IDTdna (Supplementary S1 table) were used for the amplification of the cDNA template. To ensure the correctness of the comparison, the chicken or human ACTB (β-actin gene) was used as normalizing control. The following amplification conditions were used: 95 °C for 3 min, 40 cycles of 95 °C for 15 s, 60 °C for 30 s. Sample reactions, including negative controls (MVA-GFP), were performed in triplicate and two independent repetitions. Fold change data was obtained using the delta-delta C_T_ method as previously described^17^.

#### Recombinant protein expression patterns of MVA constructs using immunoblotting and immunofluorescence

We seeded DF-1 and Vero cells at 5 × 10^5^ cells/well into six-well plates and 24 h later infected at an MOI of 5 PFU/cell in OptiMEM (Thermo Fisher Scientific, # 22600134, Waltham, MA) in the absence of FBS. At 24 h after infection, supernatants from the infected cells (∼500 µl) were centrifuged at 14,000 rpm for 25 min at RT and then concentrated to 15 µl by ultrafiltration using Microcon-3™ (Amicon Inc., Beverly, MA). The supernatants were combined with an equal volume of 2X Laemmli buffer (Bio-Rad, #1610737, Richmond, CA) and heated to 95 °C for 5 min. Infected cells were harvested and lysed using RIPA lysis buffer (Thermo Fisher Scientific, # 89900, Waltham, MA) at 4 °C for 30 min under gentle agitation. Lysates were then centrifuged for 10 min at 13,000 rpm, diluted into 4 × Laemmli buffer (Bio-Rad, Richmond, #1610747 CA), heated at 95 °C for 5 min, and 30 μl were loaded into a Bio-Rad 4–20% precast gel. Gels were transferred to a nitrocellulose membrane using Trans-Blot Turbo Blotting System following the manufacturer’s instructions. Swine serum from a convalescent animal inoculated with ASFV and anti-ß-actin antibody (Sigma, # A5441, St Louis, MO) were used to probe blots at a dilution of 1:5000. Goat anti-porcine IgG-HRP-conjugated (Southern biotech, # 6050-05, Birmingham, AL) and goat anti-mouse IgG-HRP-conjugated (Thermo Fisher Scientific, # 31430, Waltham, MA) were used to detect p30 and ß-actin, respectively. Membranes were developed using a 1-Step Ultra TMB-Blotting Solution (Thermo Fisher Scientific, #37574, Waltham, MA).

The band intensity of p30 and ß-actin were quantified by ImageJ software (version 2.1.0/1.352 k)^32^. The developed membrane was scanned with an Epson Scanner (EPSON Perfection 4490 Photo) using the Epson Scan Utility v3.24 software. The membrane was scanned at 300 pixels per inch and saved in TIFF format. Next, the obtained image was converted to a 32-bit format, and the brightness and contrast were adjusted for optimal visualization. The analysis of images was performed using the “Gel Analysis” function. Each band was selected with the rectangular region of Interest selection tool, then quantified the peak area of obtained histograms. The acquired data was normalized using ß-actin to obtain the relative quantification of the p30 protein^33^.

To perform the immunofluorescence assay, DF-1 and Vero cells in 12-well plates were infected at an MOI of 1 with each construct and MVA-GFP; an additional well was left uninfected to serve as negative infection control. After 24 h, cells were fixed with a 1:1 mix of 100% methanol and 100% acetone, washed three times with PBS, and permeabilized with a PBS/0.05% Triton X-100 solution for 15 min. The plates were then washed and blocked with 3% non-fat milk in PBS solution, and after blocking, plates were incubated with the anti-ASFV serum used in the western blot assay. Wells were washed three times for 10 min each with a PBS/1.5% 3% non-fat milk /0.05% Triton X-100 washing solution. A secondary antibody Alexa Flour 488 tagged goat anti-porcine antibody (Southern biotech, # 6050-30, Birmingham, AL) was used at a 1:2000 dilution. After washing, plates were visualized under a fluorescent microscope (AMG EVOSfl, Thermo Fisher Scientific Inc, Waltham, MA, USA).

Images taken from the immunofluorescence assay were quantified using an image processing protocol^34^. Briefly, plates were photographed at 10X objective (70% brightness) under a fluorescent microscope (AMG EVOSfl, Thermo Fisher Scientific Inc, Waltham, MA, USA). Four equally sized fields from the center of each well were selected and photographed (left to right). Images were processed through ImageJ and Fiji^35^. First, the images were transformed into an 8-bit binary representation (every pixel stored as a single bit), indicating the presence (red) and absence (black in the background) of GFP fluorescence. Then, images were processed using the Threshold option and the function “Analyze Particles” was used to count the total number of fluorescent particles per field. This command grouped and counted the red pixels with a general size area and circularity to capture every area of fluorescence (size area: 0–infinity; circularity: 1). A summary including the number of particles counted in each image was generated and used in the analysis.

#### Detection of specific anti-p30 antibodies

ELISA assay was performed as described by Brewoo et al.^27^. Briefly, 96-well ELISA plates were coated with 4 ng of p30 protein (baculovirus-expressed) diluted in 100 μl carbonate-bicarbonate buffer, pH 9.6 per well, at 4 °C overnight. Coated plates were washed three times with PBS-T (0.05% Tween-20) and incubated with blocking buffer (5% BSA in PBS) at room temperature (RT) for 1 h. Serum samples were serially diluted in triplicate from 1:50–1:1600 in blocking buffer, added to the ELISA plates, and incubated at RT for 2 h. Samples from MVA-GFP group were used as the negative control. After washing, 100 μl of a 1:5000 dilution of horseradish peroxidase (HRP)-conjugated goat anti-porcine IgG (Southern biotech, #6050-05, Birmingham, AL) was added to each well and incubated at RT for 1 h. Plates were washed six times, and 100 μl of tetra-methyl-benzidine (TMB) chromogen (Sigma, #37574, St Louis, MO) was added to each well and incubated in the dark for 5 min. The reaction was then stopped by adding 100 μl per well of 2 mM H_2_S0_4_. Absorbance was measured using an ELx800 microplate reader (BioTek, Winooski, VT) at test wavelength of 450 nm and a reference wavelength of 630 nm. The highest dilution that was positive (exceeded the mean of MVA-GFP control samples plus three standard deviations) was considered the endpoint, and its reciprocal value was transformed in geometric mean titers (GMT-log_10_) to calculate of the antibody titers of each group.

### Animal experiments

This study was carried out in strict accordance with the recommendations in the Guide for the Care and Use of Laboratory Animals of the National Institutes of Health. The IACUC protocol (Protocol #V005220) was approved by the Institutional Animal Care and Use Committee of the University of Wisconsin. Additionally, we confirm that this study is reported in accordance with ARRIVE guidelines.

Groups of five- to six-week-old female BALB/c mice (N = 4) (Harlan Sprague Dawley, Indianapolis, IN) received primary and two booster immunizations (21 and 35 days apart) with each MVA construct via intradermal injection into the hind, left footpad. A dose of 1 × 10^7^ PFU in 50 μl was used for all MVA-p30 constructs. Negative control groups were immunized with MVA-GFP at the same dose. Mice were bled via the maxillary vein at days 0, 21, 28, and 42 post-vaccination (p.v.) to monitor levels of anti-p30 antibodies.

### Statistical analysis

Statistical analyses were performed using GraphPad Prism 9 software (La Jolla, CA, USA). The significance of the difference in the p30 and mCherry mRNA expression and the anti-p30 antibody titers among the groups was determined by one-way ANOVA, followed by Tukey’s multiple-comparison test and a P-value of P < 0.05 was considered significant.

## Supplementary Information

Supplementary Information.
