# Supplementary material for: Optimization in the expression of ASFV proteins for the development of subunit vaccines using poxviruses as delivery vectors
Source: Sci Rep. 2021 Dec 6;11:23476. doi: 10.1038/s41598-021-02949-x (PMC8648923; doi:10.1038/s41598-021-02949-x)
Supplement: Supplementary file 1 — Supplementary Information. [file 41598_2021_2949_MOESM1_ESM.pdf]

Supplementary information

Optimization in the expression of ASFV proteins for the development of subunit vaccines using poxviruses as delivery vectors.

Authors

Jaime Lopera-Madrid; Lex G. Medina-Magües; Douglas P. Gladue; Manuel V. Borca; Jorge E. Osorio.

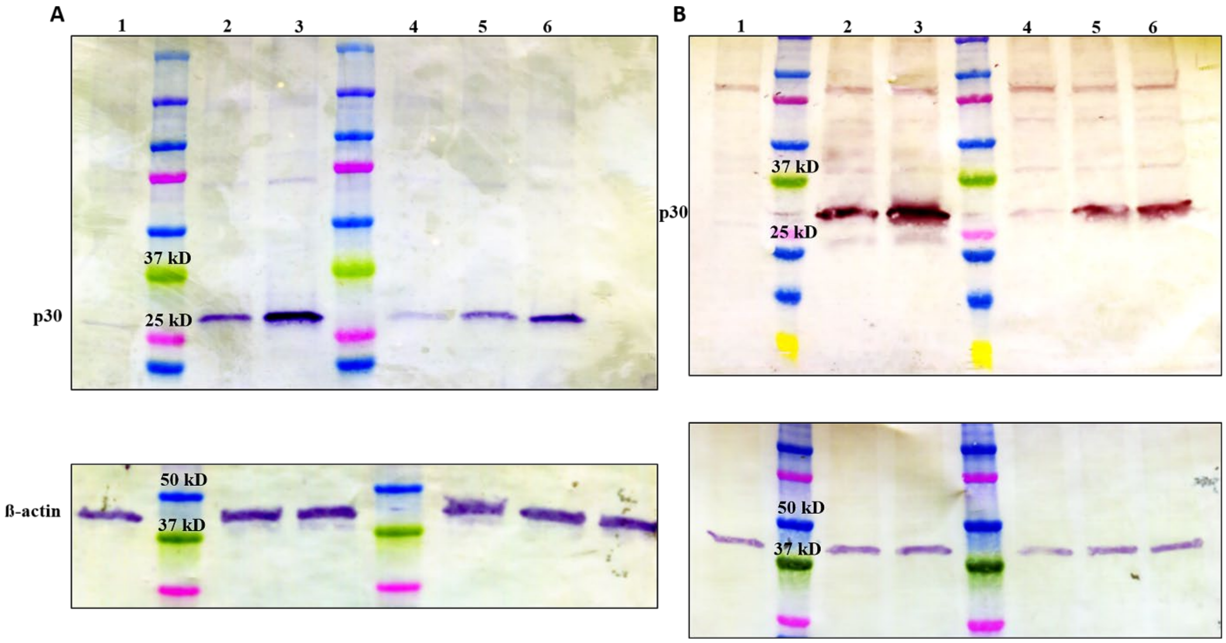

Figure S1. **Expression of p30 protein**. Monolayers of DF-1 and Vero cells were infected with each MVA-p30 virus at an MOI of 5, and then total protein extraction was performed at 24 hr p.i. Extracted proteins were subjected to SDS-PAGE followed by western blot analysis, and parallel blots were incubated with swine serum from a convalescent animal inoculated with ASFV, or an anti-ß-actin antibody (Sigma, # A544). (A) indicates proteins from DF-1 cells and (B) from Vero cells. The order is as follows, MVA-GFP control (1), PrMVA-13.5 (2), pHyb (3), S E/L-C13L (4), PrS5E (5), and S E/L (6). A parallel blot incubated with a ß-actin specific monoclonal antibody served as loading control. Molecular masses of marker proteins are indicated.

Primer name	Sequence (5' → 3')	Length	Fluorophore	Amplicon (bp)	Target
ASFV-p30_F3	CCCGACTTCAACAAGGTGAT	20	None	84	ASFV p30
ASFV-p30_R3	CACCTCCTTCTCCTCCTCCT	20	None		
ASFV-p30_P3	CGCGCCCACAACTTCATCCA	20	5' 6-FAM/ZEN/3' IBFQ		
mCherry_F1	GACCACCTACAAGGCCAAGA	20	None	79	mCherry
mCherry_R1	GTGGGAGGTGATGTCCAACT	20	None		
mCherry_P1	CCGGCGCCTACAACGTCAAC	20	5' 6-FAM/ZEN/3' IBFQ		
cACTB_F1	TGCGTGACATCAAGGAGAAG	20	None	90	*Gallus gallus* ACTB (beta-actin)
cACTB_R1	AGAGCTAGAGGCAGCTGTGG	20	None		
cACTB_P1	CGTCGCACTGGATTTCGAGCA	21	5' 6-FAM/ZEN/3' IBFQ		
hACTB	PrimeTime Std qPCR Assay - Primers and probe for Human ACTB (beta-actin). IDTdna (#Hs.PT.39a.22214847)				

Table S1. Description of primers and probes used to amplify the p30 and mCherry proteins. To ensure the correctness of the comparison, the chicken or human ACTB (β-actin gene) were used as normalizing control.
